# Soil-borne fungi challenge the concept of long-term biochemical recalcitrance of pyrochar

**DOI:** 10.1038/s41598-018-21257-5

**Published:** 2018-02-13

**Authors:** José M. De la Rosa, Ana Z. Miller, Heike Knicker

**Affiliations:** Instituto de Recursos Naturales y Agrobiología de Sevilla (IRNAS-CSIC), Av. Reina Mercedes 10, 41012 Seville, Spain

## Abstract

Pyrogenic organic matter (PyOM) is assumed to be biochemically recalcitrant, but recent studies indicated a quick decrease of PyOM in post-fire soils. Regardless erosion and abiotic degradation, microbial decomposition has been the explanation for this response, but no direct proof has been provided up to now. In the present study, we were able to demonstrate for the first time that the soil-borne fungus *Fusarium oxysporum* is not only colonizing the pore system of pyrochar (PyC) but is also involved in the degradation of its aromatic network. We showed that PyC not only stimulates microbial degradation of soil organic matter (SOM), but is also attacked and decomposed by microorganisms. Our observations are based on the chemical and morphological alterations of a sewage-sludge derived PyC produced at 600 °C after its amendment to a Calcic Cambisol by solid-state ^13^C nuclear magnetic resonance spectroscopy, analytical pyrolysis, elemental analysis, field emission scanning electron microscopy and DNA-based analysis of the isolated fungi. We showed that biofilms detected in the PyC play an essential role in the degradation process. These results are indispensable for a reliable assessment of the carbon sequestration potential of PyC in soils but also for improving global C cycling models.

## Introduction

Pyrochar (PyC), a type of pyrogenic organic matter (PyOM) generated by heating biomass residues under oxygen-limited conditions, represents a valuable strategy for land maintenance, agriculture improvement and climate change mitigation^1,2^. Its application as a soil amendment has been proposed as a mean to increase the recalcitrant soil organic matter (SOM) pool^3^, because polycondensed aromatic structures are commonly expected to have a low bioavailability^4^. Zimmerman^5^, Keith *et al*.^6^, Singh *et al*.^7^, among others, reported mean residence times (MRTs) of carbon from different biochars and charcoals in the range of centuries to millennia. This is supported with a recent meta-analysis of the biochar stability in soils by Wang *et al*.^8^, assuming that only a small part of biochar is bioavailable and that the remaining C contributes directly to long-term C sequestration in soil.

**Figure 1:**
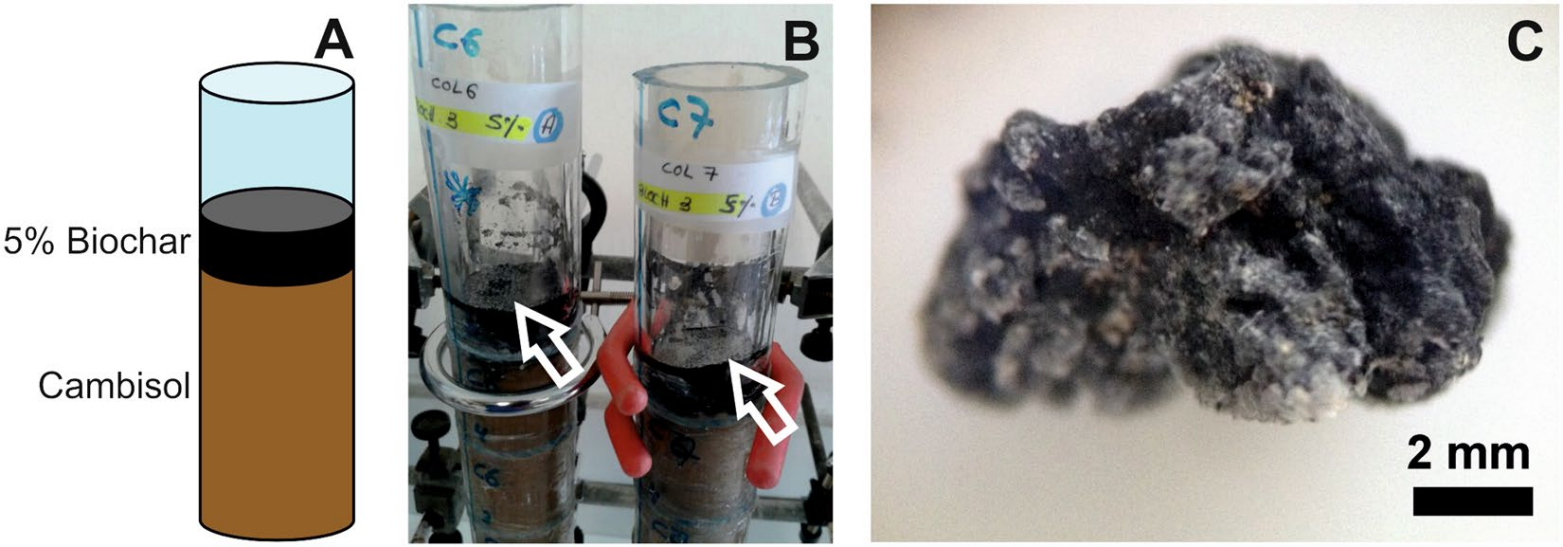
Pyrochar degradation experiment. (**A**) Schematic diagram of the columns used for the pyrochar degradation experiment. (**B**) Digital photograph of the columns showing a white coating homogeneously distributed over the pyrochar layer (arrows). (**C**) Pyrochar fragment depicting white cottony-like colonies on its surface.

On the other hand and bearing in mind the high aromaticity of this soil amendment, continuous application of this durable material may augment the aromaticity of the soil. Yet, recent studies on medium-term impact of forest fires clearly demonstrated a considerable decline of the SOM aromaticity already during the first years after the fire^9,10^. Evidences for the biochemical degradation of PyOM were recently reported by De la Rosa and Knicker^9^ and Hilscher *et al*.^11^. Instead of millennia, Hilscher *et al*.^11^, Hilscher and Knicker^12^ and De la Rosa *et al*.^13^ determined stability in the range of a few decades for natural and laboratory-produced PyC and several biochars. This shorter turnover times are certainly an advantage with respect to preventing increasing hydrophobicity and aromatization of soils treated with PyC but are also jeopardizing the concept of using PyC as a long-term C sequestration method. Schmidt *et al*.^14^ indicated that the persistence of organic carbon in soils is not primarily a molecular property, but an ecosystem feature. Early indications for the biodegradability of wood charcoals and natural coals were given by Potter^15^. Hilscher and Knicker^12^ suggested that the degradation of PyOM includes a partial oxidation of aryl structures within two steps. First, microbially accessible aryl rings are modified by substitution with OH-groups to form catechol-like structures. Subsequent cleavage of the O-aryl rings leads to an increase of carboxyl/carbonyl C. Such reactions alter the chemical and physical properties of the aging char and turn it into a substrate that is even more available for further microbial attack or adsorption. The proposed degradation pattern is likely to be related to the lignin degradation pathway performed by white rot basidiomycetes which involves the production of extracellular hydroxyl radicals (∙OH) and extracellular enzymes with an extremely high oxidative potential such as lignin peroxidase and manganese peroxidase (MnP). Considering that lignin peroxidase has broad substrate specificity for aromatic compounds, it is probable that it is also involved in the first steps of charcoal degradation. Alternatively, lignin degradation has been observed for *Ascomycetes* and *Deuteromycetes* (soft-rot fungi)^16^. A peroxidase H8 homologous to that described for *Phanerochaete chrysosporium* was identified in *Fusarium oxysporum*, which was shown to solubilize low-rank coal^17^. However, applying field emission scanning electron microscopy (FESEM) to examine the extent and nature of fungal colonization of *Pleurotus pulmonarius* and *Corolus versicolor* in biochars, Ascough *et al*.^18^ observed that during the first three months the majority of fungal growth occurred only within the top few mm of the charcoal surface. They demonstrated that the fungi growing on charcoal with no addition of glucose were actively searching for energy sources by devoting resources to rapid development of few long hyphae, but did not use the PyC as C source. Dai *et al*.^19^ found high concentration of *Actinobacteria* in charred residues extracted from incubated soils 240 days after amendment of low temperature pyrochars (350 °C) from swine manure. It was suggested that they thrive mainly on the alkyl C constituents of the char. Addition of high-temperature chars (700 °C) which were dominated by aromatic structures, considerably reduced the microbial activity of the soils, when compared with the un-amended control. This is in contrast to the finding that the saprophytic fungus *Schizophyllum commune* degrades condensed aromatic structures of charcoals^20^ and indicates that the diversity of microbial species and strategies is likely to result in a range of different responses to charcoal. Similarly, Wardle *et al*.^21^ reported that fire-derived PyOM causes loss of forest humus by stimulating the mineralization of native SOM. A comparable conclusion is reported by Wang *et al*.^8^, who stated that biochar might stimulate microbial activity especially in soils with low fertility. However, if highly aromatic structures are indeed accessible to higher extend to microbial and fungal biota, the concept of PyC application to soils as a mean for increasing the terrestrial C sink^22^ has to be re-evaluated. Therefore, we amended a Calcic Cambisol with a PyC produced at 600 °C from sewage sludge under pyrolytic (oxygen-depleted) conditions and incubated it under laboratory conditions for 120 days (Fig. 1A). Already during the first weeks of incubation, we observed white fungal colonies on the PyC layer, which we isolated and identified by DNA-based analysis. FESEM gave us information on microbe-char assemblages. We hypothesized that the observed fungal colonies obtain their energy from metabolizing PyC, which is expected to change the chemical composition of the charred residues. In order to evidence such alterations, we applied elemental analysis, solid-state ^13^C nuclear magnetic resonance spectroscopy (NMR) and analytical pyrolysis.

## Results and Discussion

### Fungi colonize pyrochar

**Figure 2:**
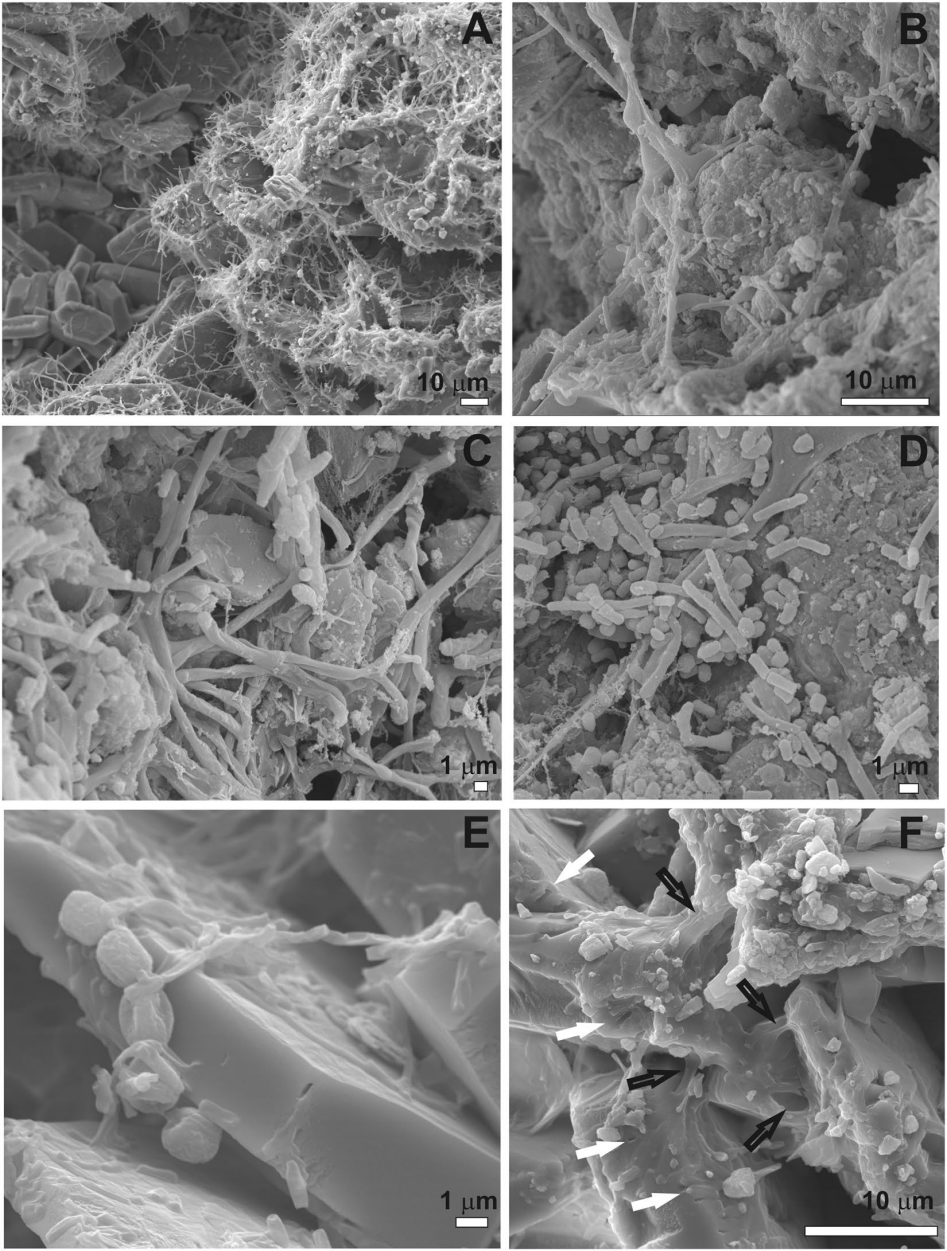
FESEM images of the pyrochar samples revealing: (**A**) abundant microbial structures coating the char particles; (**B,C**) fungal-like hyphae; (**D**) coccoid and rod-shaped cells; (**E**) fungal spores with 2–3 µm in size; (**F**) extracellular polymeric substances attached to the pyrochar (black arrows), and dissolution features on Ca-rich mineral surfaces composing the pyrochar (white arrows).

Already after 30 days of incubation, white colonies were visible to the naked eye on the surface of the PyC amended to the Cambisol. Thirty days later, this spots converted into a dense and homogeneously distributed white coating over the PyC layer, which remained visible until the end of the experiment (Fig. 1B). Under the stereomicroscope, these PyC particles showed white cottony-like colonies coating their surfaces (Fig. 1C). The FESEM images of the colonized PyC confirmed abundant microbial structures (Fig. 2A), resembling fungal hyphae (Fig. 2B,C), coccoid and rod-shaped cells (Fig. 2D), and fungal spores (Fig. 2E). In addition, extracellular polymeric substances (EPS) attached to the PyC were observed (Fig. 2F). They are excreted by microbial cells for enhancing their attachment to the substrate and protect cells from desiccation^23^. Biofilms may play an essential role for the microbial degradation of aromatic structures of PyC. Hernandez *et al*.^24^ reported that after diffusion into an active biofilm, aromatic compounds were efficiently microbial degraded, in particular toluene.

In our samples, first evidences for biodegradation of the sludge PyC were identified by the presence of dissolution features on the Ca-rich mineral surfaces of the PyC (Fig. 2F). Such features occur due to the release of organic acids that etch or solubilize the mineral substrate^25–27^. Although it is difficult to determine the exact mechanism of microbial corrosion, the pitting of PyC mineral grains, as shown in Fig. 2F, clearly demonstrates the occurrence of microbial-induced degradation in the incubated PyC.

**Figure 3:**
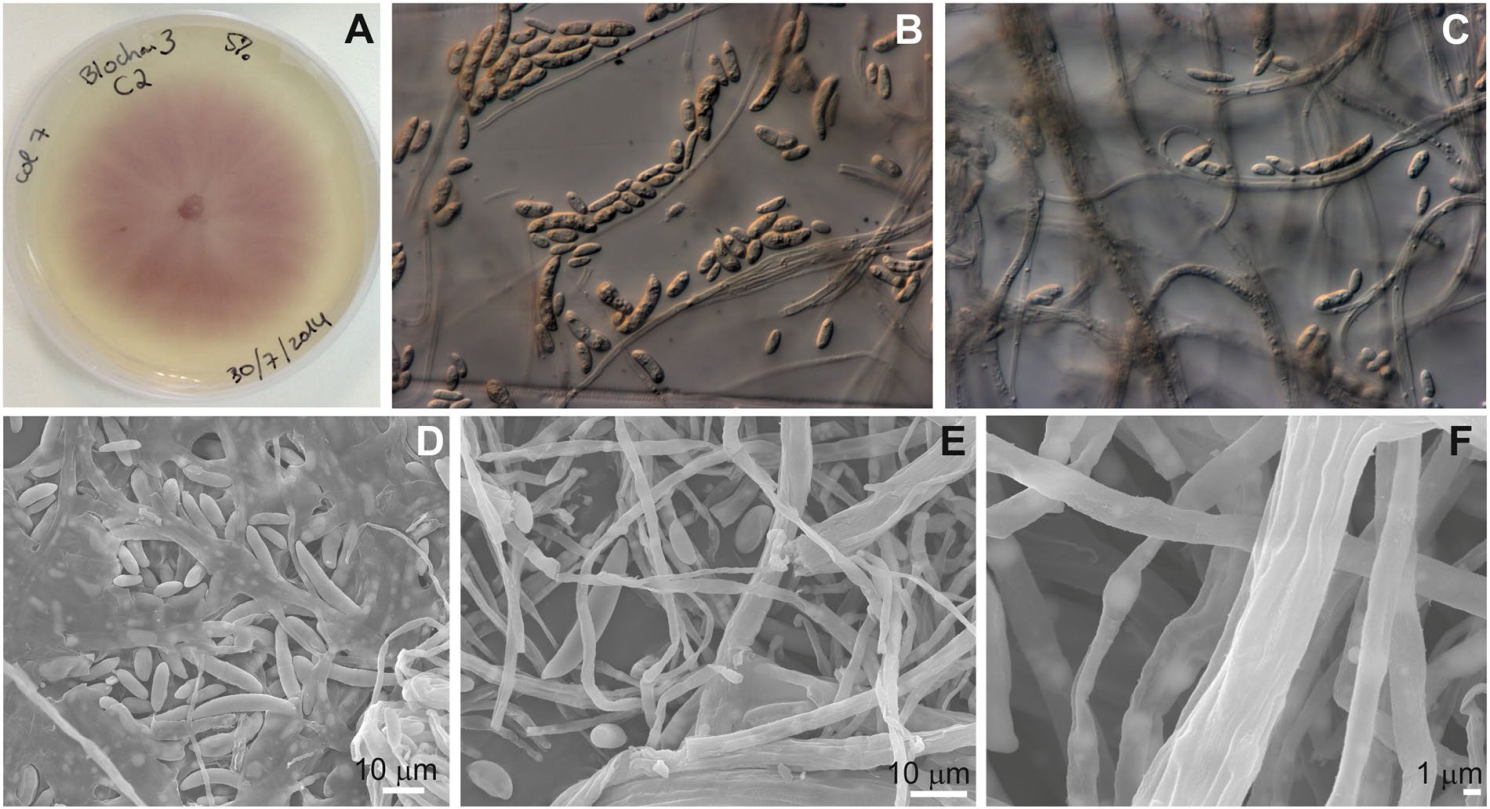
Fungal isolates obtained from the pyrochar particles. (**A**) Fungal colony morphology and pigmentation on MEA medium after 5 days of incubation at 22 °C. (**B,C**) Light microscope image showing abundant microconidia and macroconidia, characteristic of *Fusarium* sp. (**D**) FESEM image of the fungal mycelium showing micro and macroconidia. (**E**) FESEM image of the fungal hyphae and a macroconidium. (**F**) FESEM image of chlamydospores.

Three fungal isolates were collected from PyC particles exhibiting white cotton-like colonies on their surface. Colony morphology on malt extract agar (MEA) culture medium were similar for the three isolates, showing initially white aerial cottony mycelium, which become tinged with pink salmon and purple after 4–5 days of growth (Fig. 3A). Under the light microscope, abundant microconidia, macroconidia and chlamydospores were identified (Fig. 3B,C). The microconidia were one or two-celled, generally oval or kidney-shaped, but spherical were also observed (Fig. 3B). Macroconidia were slightly curved, generally with one or two septa (Fig. 3C). Examination of the isolated fungi by FESEM was in line the light microscopy results, showing the one-celled, oval-shaped microconidia and slightly curved macroconidia (Fig. 3D). Abundant hyphae (Fig. 3E) and intercalary chlamydospores, mostly single but sometimes in pairs, were clearly observed by FESEM (Fig. 3F). According to the macroscopic morphology of the colonies and the microscopic features, the fungal isolates are morphologically compatible with *Fusarium* sp.^28,29^.

**Figure 4:**
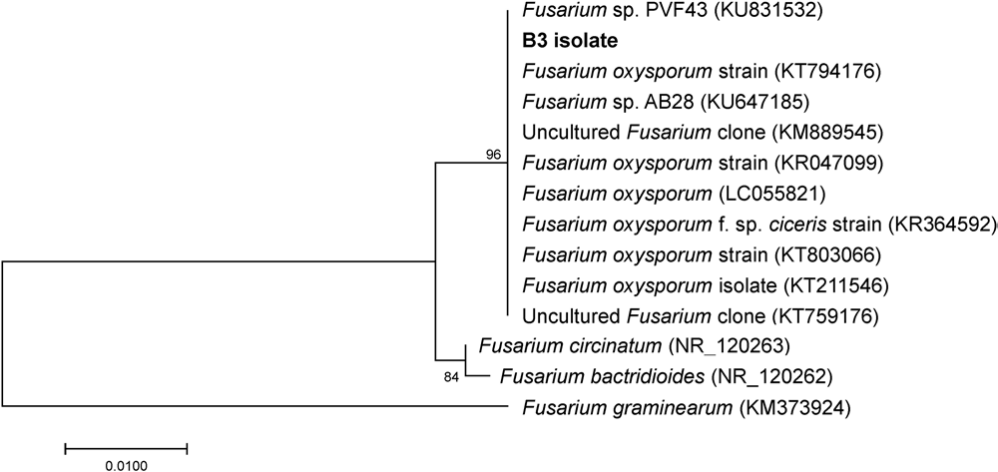
Phylogenetic tree derived from ITS1–5.8S-ITS2 regions of rRNA gene sequences showing the relationships between the isolated fungal strain (B3 isolate) and the closest related sequences. The tree was inferred using the Neighbor-Joining method.

The comparative analyses of the ribosomal internal transcribed spacer (ITS) sequences of the three isolates showed to be identical (100% similarity among sequences). The identified fungus grouped into the *Fusarium* genus within the Ascomycota phylum and was affiliated to the *Fusarium oxysporum* species complex, with 100% similarity with their closest relatives obtained from both NCBI and SILVA databases. A Neighbor-joining phylogenetic tree was created using *F*. *oxysporum* ITS sequences available in the SILVA database, which showed that the isolated fungus and closest relatives were classified into one clade supported by high bootstrap value (Fig. 4). The closest cultured matches were recovered from plant leafs and roots. In addition, the isolated strain claded with two uncultured *Fusarium* clones (KM889545 and KT759176) from agricultural and rhizosphere soils, respectively. These fungi are widespread in all types of soil and are common soil saprophytes^30^. *F. oxysporum* strains are known to degrade lignin, complex carbohydrates associated with soil debris^16,31^ but also polycyclic aromatic hydrocarbons (PAHs) both under oxic^32^ and microaerobic conditions^33^. These authors suggested that soil fungi are capable of growth on PAHs as primary carbon source.

### Chemical alterations of biodegraded pyrochar

The original sludge PyC contained 187 g C and 20 g N per kg material (Table 1). Comparable values were reported previously for other sewage sludge chars^34^. The relatively high N content is best explained by the thermally induced transformation of peptide-like compounds into N-heteraromatic structures^35^. The PyC degradation experiment resulted in a reduction of the C content (from 187 ± 7 to 164 ± 5 g C kg^−1^), whereas the relative content of N increased. Thus, the C/N ratios decreased sharply from 9.4 to 5.9. This is extremely low if compared with wood-derived PyC typically applied to soils^36^. Although these figures fall within the optimal range of soil microbial C/N ratio for soil productivity^37^, it is likely that inorganic N accumulated during the experiment. After 120 days, the atomic (at) H/C ratio increased from 1.0 to 1.7, which is attributable to an increase of the protonation with a concomitant reduction of the condensation degree of the PyC. The O/C_at_ slightly increased (from 0.4 to 0.5) which may indicate the introduction of carboxylic groups during the oxidation of aromatic ring structures as it was observed during the degradation of grass char^12^.

**Table 1:** Elemental (C, H, N, O) and physicochemical properties of the pyrochar before and after 120 days of incubation.

Sample	C (g kg^−1^)	H (g kg^−1^)	N (g kg^−1^)	O (g kg^−1^)	H/C_at_	O/C_at_	C/N	pH	WHC^a^ (%)	Ash Content (750 °C) (%)
Sludge pyrochar (t0)	187 ± 7	15 ± 3	20 ± 2	92 ± 11	1.0	0.4	9.4	6.7 ± 0.2	27 ± 5	69 ± 1.2
Incubated sludge pyrochar (120 days)	164 ± 5	23 ± 4	28 ± 5	112 ± 24	1.7	0.5	5.9	6.9 ± 0.1	25 ± 8	67 ± 0.9

^a^Water Holding Capacity (%).

**Figure 5:**
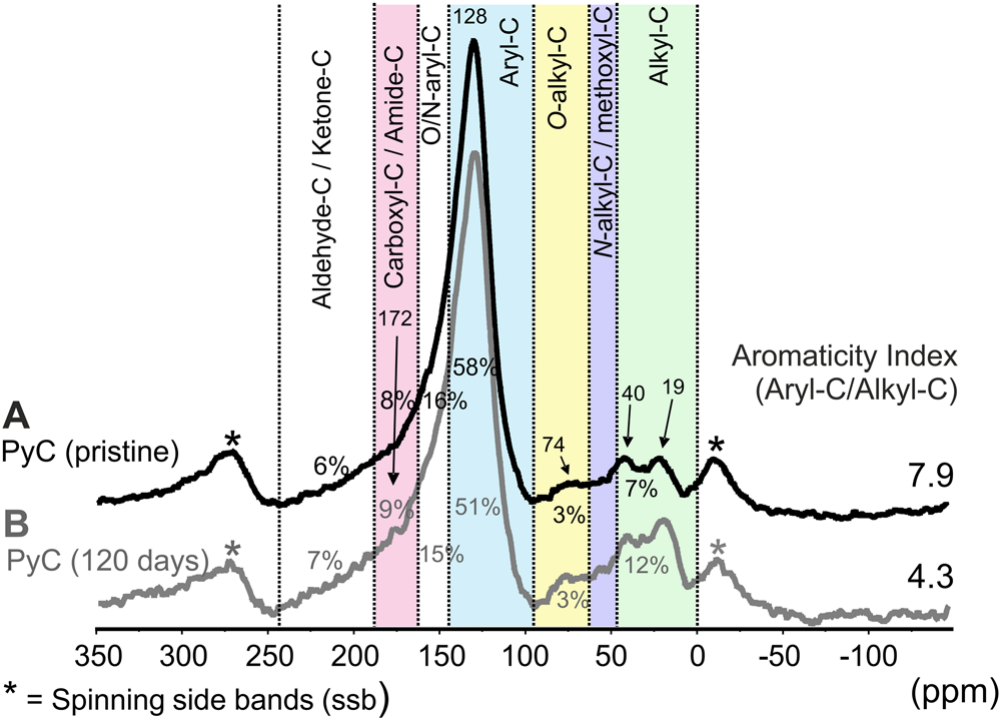
Solid state ^13^C NMR spectra and ^13^C intensity distribution over each chemical shift regions (in ppm) by the integration routine with MestReNova 10 Software. (**A**) Pristine sludge pyrochar. (**B**) Pyrochar after 120 days of incubation under controlled conditions.

Figure 5 shows the ^13^C NMR spectra of the PyC and its intensity distribution. Aryl C (140–90 ppm) represents the dominating C form in both samples. The spectrum of the pristine sludge PyC exhibits 58% of its total ^13^C intensity within this chemical shift region (Fig. 5A). For the incubated PyC, this C type decreases to 51% of the total C (Fig. 5B). Taking into account the elemental composition of both PyC samples and the differences in the relative abundance of aryl C, we can estimate that approximately 1.6 g of aryl C kg^−1^ were degraded in a period of 4 months. The chemical shift region from 160–140 ppm covers the resonance lines of heteroaromatic C. Considering the low intensity in the chemical shift region of N-alkyl C (60–45 ppm), the high N content of both samples suggests high contributions of N heterocyclic aromatic C. The contribution of the alkyl C region (45–0 ppm) to the total ^13^C intensity increased from 7% to 12% after the laboratory-based experiment. Thus, the aromaticity of the PyC decreased from 7.9 to 4.3, possibly due to the accumulation of microbial residues within the PyC. The other regions remained practically unaltered. In the region corresponding to carboxyl/amide C (185–160 ppm), a slight shoulder at 172 ppm is visible for the colonized PyC (Fig. 5B). This could be an indication of oxidized aromatic rings, but may also be caused by ester and amides in the growing biomass. The lack of higher carboxylic C contents after degradation may be explained by a more or less degradation of the aromatic breakdown products caused by fungi, including *F. oxysporum*, which can degrade lignin using ligninolyitic extracellular peroxidases and laccases^38^. A fast degradation of the aromatic breakdown products through the β-ketoadipate pathway and the formation of acetyl-CoA may be responsible for the lack of higher carboxylic C accumulation^39^. The ^13^C NMR spectra confirmed the results of elemental (H/C_at_) and microscopy analyses. The development of native soil microbial community on the PyC layer, in particular of *F*. *oxysporum*, is causing a notable reduction in the aromaticity of the tested sludge PyC.

**Figure 6:**
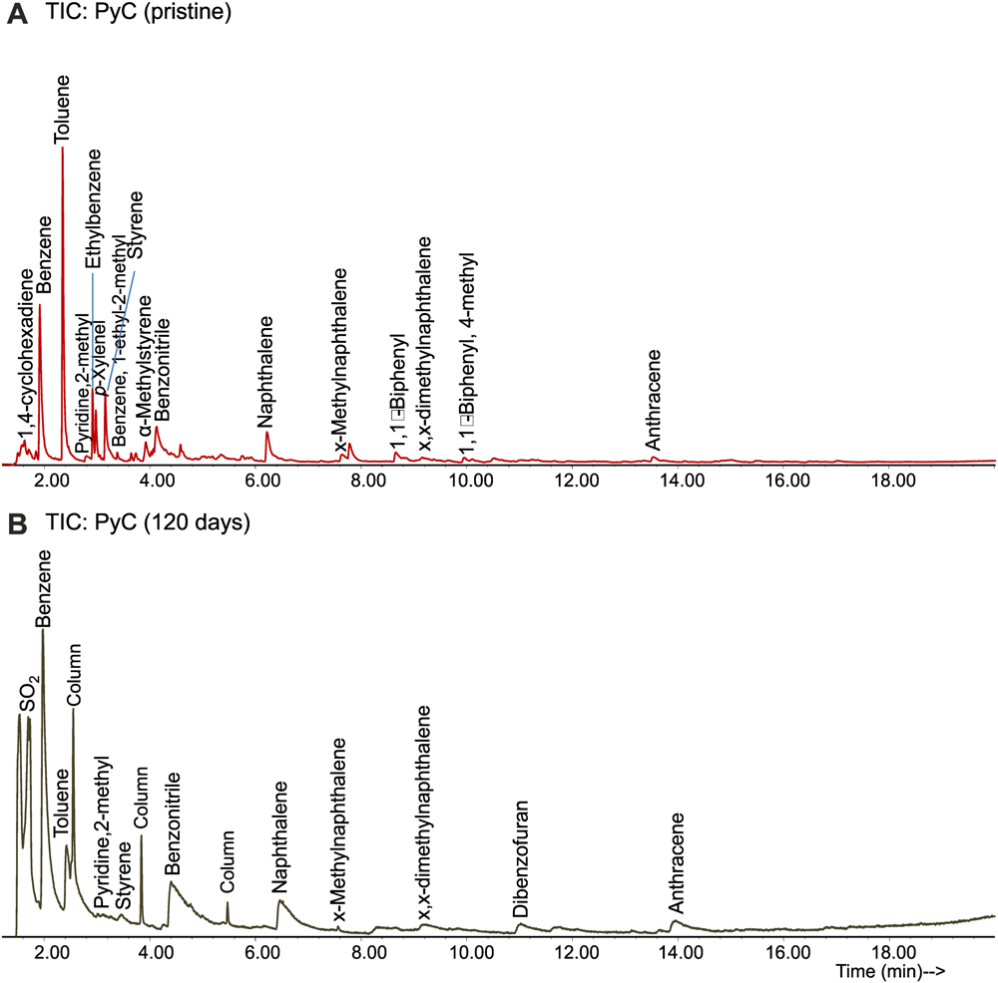
Pyro-chromatograms of the sludge pyrochar samples and compound identification by pyrolysis gas chromatography-mass spectrometry. (**A**) Pristine sludge pyrochar. (**B**) Pyrochar after 120 days of incubation under controlled conditions.

Product distribution corresponding to the total ion chromatograms (TIC) of the pristine and hand-picked colonized PyC particles after 120 days of incubation are shown in Fig. 6. The relatively low abundance of pyrolyzable aromatic homologues is best explained by the fact that the pyrolysis temperature (600 °C) for PyC production is similar to the pyrolysis temperature for analytical pyrolysis, which means that the volatile products have been already released. The pyro-chromatograms of both samples are very similar, being dominated by benzene, toluene and polycyclic aromatic compounds (Fig. 6A,B). Furthermore, the presence of N-containing compounds, such as pyridine or benzonitrile, is related to the nature of the N-rich feedstock. However, when applying solid-state ^15^N NMR spectroscopy to PyOM those compounds were not identified to a higher extent^35^. The pyro-chromatogram of the PyC after 120 days of incubation indicates microbial growth by showing some differences (Fig. 6B). The decrease in the ratio toluene/benzene points to an alteration of the PyC. Wan *et al*.^40^ and Gopinath and Dhanasekar^41^ reported efficiencies of up to 100% by bacteria and fungi for the removal of toluene. Similarly, the lack of biphenyl compounds in the incubated PyC could suggest a preferential biodegradation of these compounds.

### Impact of fungal degradation of pyrochar on terrestrial C storage

We obtained clear evidence that *F*. *oxysporum* develop on PyC obtained from N-rich sewage sludge. We were able to show that this soil-borne fungus also forms biofilms within the charred core and that they degrade PyC without additional stimulation. Thus, as known to the authors, we demonstrated for the first time that those microorganisms are not only using the pore system of the PyC as habitat, but are indeed able to feed on aromatic C in the condensed network of this material. It is likely that the biofilm plays an important role as a matrix for the microorganisms but also for the activity of the extracellular lignolytic enzymes. The degradation may have been supported by the high N- and P-contents of our pyrolyzed sewage sludge, which provided the necessary micronutrients for the build-up of new biomass. However, if the availability of micronutrients represents an important requirement for the degradability of PyC is still an open question. Future experiments with nutrient poor PyC may help to clarify this issue.

Our observations have a major impact on the concept of the stability of PyC and biochar in soils. As it can be biochemically attacked by soil-borne fungi, its longevity is likely to be much shorter as previously assumed. Consequently, its potential for C sequestration in soils may have been overestimated. On the other hand, this does not imply that soil amendment with biochar or other carbonized organic matter represents a senseless approach if augmentation of the slow carbon pool is wanted. In contrary, since recent studies indicated PyC turnover rates in the range of humified organic residues, the added pyrogenic C will directly enter the slow carbon pool without providing new material, which is easily available for a quick transformation into CO_2_. Here one has to bear in mind that production of PyC includes energy and CO_2_ release during its formation, but modern devices and processes are available to use the pyrolysis process for a net-energy production. Considering further that deposition of biowaste in landfills is associated with high losses of N and P due to mineralization and subsequent leaching or in the case of N also by volatilization, application of biochar and other sustainable carbonized organic materials certainly remains a valuable approach for a responsible use of resources by concomitantly decreasing greenhouse gas emissions.

## Methods

### Production of pyrochar

The PyC was produced from sewage sludge (75% dry matter), with a PYREG® 500 – III pyrolysis units for 20 minutes at a temperature of 600 °C (PYREG GmbH, Dörth, Germany) by the PYREG GmbH. No inert gas was used to drive off pyrolytic vapors. A detailed description of the pyrolysis conditions and the composition of the PyC are given in De la Rosa *et al*.^42^. In this paper, the term biochar has been intentionally avoided due to the low content of organic C of the pyrolyzed sample (<500 g kg^−1^; European Biochar Certificate^43^).

### Laboratory-based degradation experiment

The soil matrix for the degradation experiment was obtained from the A_h_ horizon of a sandy loamy Calcic Cambisol (IUSS Working Group WRB, 2007), located at Coria del Río, Seville (SW Spain; 37° 21.32′ N, 6° 4.07′ W). Before the experiment, the soil was dried at 40 °C during 48 h and sieved (<2 mm). Small branches, fresh mosses and plant remains, as well as roots were removed manually. Elemental composition and physical properties of this soil are described in De la Rosa *et al*.^42^.

For the experiment two cylindrical columns with a height of 25 cm were filled with 200 g of the soil matrix and topped with 10 g of the PyC (Fig. 1A). Additionally, two columns without any PyC amendment were used as control. After adjusting the soil humidity to 60% of the maximum water holding capacity (WHC), the columns were placed into a hood at 25 °C under 12 h dark–light cycles for 120 days at the laboratory. No mineral nutrient solution or microbial culture was added. The columns were weighted daily and water content was adjusted to maintain the WHC. After 120 days of incubation, PyC particles were carefully hand-picked for analyses.

### Isolation and identification of fungi by DNA-based analysis

White colonies from both columns containing sludge PyC particles were directly plated on malt extract agar (MEA) using a sterile loop, and incubated at 22 °C in the dark for 7 days. After incubation, colonies were removed from the agar surface using a sterile loop and transferred to fresh MEA medium for further molecular identification. Three isolates were used for light microscopy and molecular analysis.

Genomic DNA from pure cultures was extracted by scraping the mycelium from the plates and transferring it to 1.5 ml Eppendorf tubes containing 350 μl of lysing buffer (TNE) and glass beads. The mixture was shaken in a cell disrupter (Fast Prep-24, Solon, OH, USA) through 3 cycles at 4.5 m/s for 30 s. The DNA was purified by phenol/chloroform extraction and ethanol precipitation. Amplifications of fungal internal transcribed spacer (ITS) regions were attempted using the ITS1 (5′-TCC GTA GGT GAA CCT GCG G) and ITS4 (5′-TCC TCC GCT TAT TGA TAT GC-3′) primers as described previously^26,44^. The PCR reactions were performed in a BioRad thermal cycler (BioRad, Hercules, CA, USA) using the following thermocycling program: 2 min denaturing step at 95 °C, followed by 35 cycles of denaturing (95 °C for 15 s), annealing (55 °C for 15 s) and elongation (72 °C for 2 min). A final elongation step of 10 min at 72 °C was added at the end. After purification, the amplification products were sequenced by MACROGEN Europe Sequencing Services (Amsterdam, The Netherlands).

Bioinformatic tools were used to infer taxonomic classification and phylogenetic tree. The three DNA sequences obtained were edited using Bioedit v7.0.4 software (Technelysium, Tewantin, Australia). After quality control, similarity search was performed using the BLASTn (Basic Local Alignment Search Tool) algorithm^45^ from the NCBI (National Centre for Biotechnology Information, http://www.ncbi.nlm.nih.gov/) and SILVA databases. One sequence of the identical isolates was deposited in GenBank with accession number LT703312. A Neighbor-Joining phylogenetic tree was constructed from the strain sequence and reference sequences using MEGA6^46^ by heuristic search. Closest relatives (with minimum identity 80%) were collected from SILVA as reference sequences for creating the phylogenetic tree. Bootstrap values were generated from 100 replicates.

### Morphological characterization

The microscopic analysis of PyC fragments (~5–6 mm) was performed with a Motic DM143 digital stereomicroscope (Motic Xiamen, China), whereas the morphology of the three individual fungal isolates were examined under a Zeiss Axioskop light microscope (Zeiss, Germany). Images were analyzed using the AxioVision Software (Zeiss, Germany).

Pyrochar samples with white colonies and without evidence of microbial growth were analyzed by field emission scanning electron microscopy. The colonized particles were fixed with 2.5% glutaraldehyde in 0.1 M cacodylate-buffer (pH 7.4) at 4 °C for 2 h. Subsequently, they were washed three times in cacodylate-buffer for 5 min each and postfixed in 1% osmium tetroxide for 1 h at 4 °C. After dehydration by subsequent dilution series in ethanol and acetone, the samples were dried in a critical point drying device (EM CPD 300, Leica) at 34.5 °C and sputter-coated with a thin gold/palladium film for their examination using a Jeol JSM-7001F microscope equipped with an Oxford X-ray energy dispersive spectroscopy (EDS) detector for elemental characterization. Non-fixed PyC particles with no evidence of microbial growth were directly mounted on SEM sample stubs and sputtered with gold/palladium. The FESEM examinations were performed in secondary electron detection mode with an acceleration potential of 15 kV.

### Analytical characterization of pyrochar

Elemental analysis (C, H, N, O) of the dry PyC was conducted in triplicate by dry combustion (1000 °C) using a Perkin-Elmer 2400 series 2 elemental analyzer.

Pyrolysis-gas chromatography–mass spectrometry (Py-GC/MS) analyses were performed in a double shot pyrolyzer (model 2020i, Frontier Laboratories) directly connected to an Agilent 6890 GC-MS system. This pyrolyzer allows the sequential examination of the products released by thermal cracking of the sample. Samples (1 mg) were placed in small crucible capsules, introduced into a preheated micro-furnace and pyrolysis was carried on for 1 min at 600 °C. The GC/MS conditions were similar to those established by De la Rosa *et al*.^47^ for the study of PyOM in soils.

Solid-state ^13^C NMR spectra of the PyC material before and after the degradation experiment were obtained with a Bruker Avance III HD 400 MHz spectrometer operating at a ^13^C frequency of 100.64 MHz and spinning ZrO_2_ rotors of 4 mm OD with Kel-F caps at the magic angle with a speed of 14 kHz. The cross polarization (CP) was achieved during a contact time of 1 ms with a ramped ^1^H pulse. Employing a pulse delay of 500 ms, over 50,000 scans were accumulated. The ^13^C chemical shifts were calibrated relative to tetramethylsilane (0 ppm) with glycine (COOH at 176.08 ppm). The spectra were quantified by integration of the following chemical shift regions: alkyl C (0–45 ppm); *N*-alkyl/methoxyl C (45–60 ppm); *O*-alkyl C (60–90 ppm); aromatic C (90–140 ppm); O/N-aryl-O C (140–160 ppm); carbonyl/amide C (160–245 ppm), using the MestreNova 10 software (Santiago de Compostela, Spain).

### Accession codes

Sequence was deposited in GenBank with accession number LT703312.

## References

[1] Woolf D, Amonette JE, Street-Perrott FA, Lehmann J, Joseph S (2010). Sustainable biochar to mitigate global climate change. Nat. Commun..

[2] Abiven S, Schmidt MWI, Lehmann J (2014). Biochar by design. Nat. Geosci..

[3] Hagemann N (2017). Organic coating on biochar explains its nutrient retention and stimulation of soil fertility. Nat. Commun..

[4] Lehmann J. & Joseph S. Biochar systems. In: *Biochar for Environmental Management: Science and Technology* (eds Lehmann, C. J. & Joseph, S.). (Earthscan, London, 2009).

[5] Zimmerman A (2010). Abiotic and microbial oxidation of laboratory-produced black carbon (biochar). Env. Sci. Tech..

[6] Keith A, Singh B, Singh. BP (2011). Interactive priming of biochar and labile organic matter mineralization in a smectite-rich soil. Environ. Sci. Technol..

[7] Singh BP, Cowie AL, Smernik RJ (2012). Biochar carbon stability in a clayey soil as a function of feedstock and pyrolysis temperature. Environ. Sci. Technol..

[8] Wang J, Xiong Z, Kuzyakov Y (2016). Biochar stability in soil: meta-analysis of decomposition and priming effects. GCB Bioenergy.

[9] De la Rosa JM, Knicker H (2011). Bioavailability of N released from N-rich pyrogenic organic matter: An incubation study. Soil Biol. Biochem..

[10] Lopez-Martin M, Velasco-Molina M, Knicker H (2016). Variability of the quality and quantity of organic matter in soil affected by multiple wildfires. J. Soils Sediments.

[11] Hilscher A, Heister K, Siewert C, Knicker H (2009). Mineralisation and structural changes during the initial phase of microbial degradation of pyrogenic plant residues in soil. Org. Geochem..

[12] Hilscher A, Knicker H (2011). Degradation of grass-derived pyrogenic organic material, transport of the residues within a soil column and distribution in soil organic matter fractions during a 28 month microcosm experiment. Org. Geochem..

[13] De la Rosa JM, Rosado M, Paneque M, Miller AZ, Knicker H (2018). Effects of aging under field conditions on biochar structure and composition: Implications for biochar stability in soils. Sci. Total Environ..

[14] Schmidt MWI (2011). Persistence of soil organic matter as an ecosystem property. Nature.

[15] Potter MC (1908). Bacteria as agents in the oxidation of amorphous carbon. Proc. R. Soc. Lond..

[16] Rodriguez A (1996). Degradation of natural lignins and lignocellulosic substrates by soil-inhabiting fungi imperfecti. FEMS Microbiol. Ecol..

[17] Mönkemann H, Hölker U, Golubnitchaya-Labudová O, Lichtenberg-Fraté H, Höfer M (1996). Molecular evidence of a lignin peroxidase H8 homologue in *Fusarium oxysporum*. Folia Microbiol..

[18] Ascough PL, Sturrock CJ, Bird MI (2010). Investigation of growth responses in saprophytic fungi to charred biomass. Isotopes Environ Health Stud. Mar..

[19] Dai Z (2017). DNA extraction efficiency from soil as affected by pyrolysis temperature and extractable organic carbon of high-ash biochar. Soil Biol. Biochem..

[20] Wengel M, Kothe E, Schmidt CM, Heide K, Gleixner G (2006). Degradation of organic matter from black shales and charcoal by the wood-rotting fungus *Schizophyllum commune* and release of DOC and heavy metals in the aqueous phase. Sci. Total Environ..

[21] Wardle DA, Nilsson MC, Zackrisson O (2008). Fire-derived charcoal causes loss of forest humus. Science.

[22] Lehmann J, Gaunt J, Rondon M (2006). Bio-char sequestration in terrestrial ecosystems - A review. Mitig. Adapt. Strat. Gl..

[23] Costerton J. W. The biofilm primer. (Springer-Verlag Berlin Heidelberg) 199 p. (2007).

[24] Hernandez, A. *et al*. Methyl tert-butyl ether (MTBE) elimination by cometabolism: laboratory and biofilter pilot-scale results. Air & Waste Management Association’s 94th Annual meeting & Exhibition. Paper 1037 (2001).

[25] Miller AZ (2010). Laboratory-induced endolithic growth in calcarenites: biodeteriorating potential assessment. Microb. Ecol..

[26] Saiz-Jimenez C, Miller AZ, Martin-Sanchez PM, Hernandez-Marine M (2012). Uncovering the origin of the black stains in Lascaux Cave in France. Environ. Microbiol..

[27] Riquelme C (2015). Actinobacterial diversity in volcanic caves and associated geomicrobiological interactions. Front. Microbiol..

[28] Leslie, J. F., Summerell, B. A. & Bullock, S. The *Fusarium* laboratory manual. 1 ed. (Wiley- Blackwell, 388 p. 2006).

[29] Ciampi L (2009). Identification of two species of *Fusarium* link that cause wilting of colored callas (*Zantedeschia aethiopica* (L.) Spreng.) cultivated under greenhouse conditions in Chile. Chil. J. Agr. Res..

[30] Burgess, L. W., Summerell, B. A., Bullock, S., Gott, K. P. & Backhouse, D. Laboratory Manual for *Fusarium* Research. 3rd ed. (University of Sydney and Botanic Garden; Sydney, Australia, 1994).

[31] Sutherland JB, Pometto AL, Crawford DL (1983). Lignocellulose degradation by *Fusariu*m species. Can. J. Bot..

[32] Kadri T (2017). Biodegradation of polycyclic aromatic hydrocarbons (PAHs) by fungal enzymes: A review. J. Environ. Sci..

[33] Silva IS, Grossman M, Durrant LR (2009). Degradation of polycyclic aromatic hydrocarbons (2–7 rings) under microaerobic and very-low-oxygen conditions by soil fungi. Int. Biodeter. Biodegr..

[34] Zhao L, Cao X, Masek O, Zimmerman A (2013). Heterogeneity of biochar properties as a function of feedstock sources and production temperatures. J. Haz. Mat..

[35] Knicker H (2010). “Black nitrogen” - an important fraction in determining the recalcitrance of charcoal. Org. Geochem..

[36] Paneque M (2016). H. Effect of biochar amendment on morphology, productivity and water relations of sunflower plants under non-irrigation conditions. Catena.

[37] Li Y (2016). Soil microbial C:N ratio is a robust indicator of soil productivity for paddy fields. Sci. Rep..

[38] Bugg TDH, Ahmad M, Hardiman EM, Rahmanpour R (2011). Pathways for degradation of lignin in bacteria and fungi. Nat. Prod. Rep..

[39] Harwood CS, Parales RE (1996). The β-ketoadipate pathway and the biology of self-identity. Annu. Rev. Microbiol..

[40] Wan Z, Chauncey DJ, Thomas MK, John PY, David M (2003). Vinyl aryl ethers from copper-catalyzed coupling of vinyl halides and phenols. Tetrahedron Lett..

[41] Gopinath M, Dhanasekar R (2012). Microbial degradation of toluene. Afr. J. Biotechnol..

[42] De la Rosa JM, Paneque M, Miller AZ, Knicker H (2014). Relating physical and chemical properties of four different biochars and their application rate to biomass production of Lolium perenne on a Calcic Cambisol during a pot experiment of 79 days. Sci. Total Environ..

[43] European Biochar Certificate, European Biochar Foundation - Guidelines for a Sustainable Production of Biochar. European Biochar Foundation, Version 6.3E of 14th August 2017. 1–24 (2012).

[44] White, T. J., Bruns, T., Lee, S. & Taylor, J. Amplification and direct sequencing of fungal ribosomal RNA genes for phylogenetics. in: (eds. Innis, M. A., Gelfand, D. H., Sninsky, J. J., White, T. J.) *PCR Protocols: A Guide to Methods and Applications*. (Academic Press, New York, pp. 315–322, 1990).

[45] Altschul SF, Gish W, Miller W, Myers EW, Lipman DJ (1990). Basic Local Alignment Search Tool. J Mol. Biol..

[46] Tamura K, Stecher G, Peterson D, Filipski A, Kumar S (2013). MEGA6: Molecular evolutionary genetics analysis version 6.0. Mol. Biol. Evol..

[47] De la Rosa JM (2012). Characterization of wildfire effects on soil organic matter using analytical pyrolysis. Geoderma.

